# A Retrospective View of the Triple-Negative Breast Cancer Microenvironment: Novel Markers, Interactions, and Mechanisms of Tumor-Associated Components Using Public Single-Cell RNA-Seq Datasets

**DOI:** 10.3390/cancers16061173

**Published:** 2024-03-16

**Authors:** Minsoo Kim, Wonhee Yang, Dawon Hong, Hye Sung Won, Seokhyun Yoon

**Affiliations:** 1 Department of Computer Science, College of SW Convergence, Dankook University, Yongin 16890, Republic of Korea; minsoo0423@dankook.ac.kr; 2 Department of AI-Based Convergence, College of SW Convergence, Dankook University, Yongin 16890, Republic of Korea; yangwonhee@dankook.ac.kr; 3 RNA Cell Biology Laboratory, Graduate Department of Bioconvergence Engineering, Dankook University, Yongin 16890, Republic of Korea; 12171520@dankook.ac.kr; 4 Department of Internal Medicine, College of Medicine, The Catholic University of Korea, Seoul 06591, Republic of Korea; 5 Department of Electronics & Electrical Engineering, College of Engineering, Dankook University, Yongin 16890, Republic of Korea

**Keywords:** breast cancer, triple-negative breast cancer, single-cell RNA-seq, tumor microenvironment, potential treatment target

## Abstract

**Simple Summary:**

Triple-negative breast cancer (TNBC) comprises about 15% of all breast cancers worldwide and is known to have a poorer prognosis compared to other subtypes of breast cancer. It poses challenges due to the absence of treatment targets. In this work, we tried to mine new markers and treatment targets using public single-cell RNA-seq datasets. We identified twelve TNBC markers along with various markers of other components around the tumor microenvironment. Among them, we noticed the *DSC2* gene, which interacts with *DSG2*, and identified its role and characteristics in TNBC. The marker can be considered as a new treatment target for TNBC.

**Abstract:**

Triple-negative breast cancer (TNBC) is a significant clinical challenge due to its aggressive nature and limited treatment options. In search of new treatment targets, not only single genes but also gene pairs involved in protein interactions, we explored the tumor microenvironment (TME) of TNBC from a retrospective point of view, using public single-cell RNA sequencing datasets. A High-resolution Cell type Annotation Tool, HiCAT, was used first to identify the cell type in 3-level taxonomies. Tumor cells were then identified based on the estimates of copy number variation. With the annotation results, differentially expressed genes were analyzed to find subtype-specific markers for each cell type, including tumor cells, fibroblast, and macrophage. Cell–cell interactions were also inferred for each cell type pair. Through integrative analysis, we could find unique TNBC markers not only for tumor cells but also for various TME components, including fibroblasts and macrophages. Specifically, twelve marker genes, including *DSC2* and *CDKN2A*, were identified for TNBC tumor cells. Another key finding of our study was the interaction between the *DSC2* and *DSG2* genes among TNBC tumor cells, suggesting that they are more tightly aggregated with each other than those of other subtypes, including normal epithelial cells. The overexpression of *DSC2* in TNBC and its prognostic power were verified by using METABRIC, a large bulk RNA-seq dataset with clinical information. These findings not only corroborate previous hypotheses but also lay the foundation for a new structural understanding of TNBC, as revealed through our single-cell analysis workflow.

## 1. Introduction

Breast cancer is a highly heterogeneous disease that involves complex molecular networks [1]. Precise classification and treatment strategies are essential due to the diverse nature of these tumors [2]. Based on the expression of estrogen receptor (ER), progesterone receptor (PR), and human epidermal growth factor receptor 2 (HER2), breast cancer is categorized into several subtypes, including hormone receptor (HR)-positive, HER2-positive, and triple-negative breast cancer (TNBC) [3]. Each subtype requires tailored treatment strategies, such as endocrine therapies for HR-positive breast cancer and targeted therapies, such as trastuzumab [4] and pertuzumab for HER2-positive breast cancer [5]. However, TNBC patients, which lack the expression of ER, PR, and HER2, have limited treatment options and poor prognoses [6,7].

Recently, immune checkpoint inhibitors such as PD-1 and PD-L1 inhibitors have emerged as promising approaches for treating patients with TNBC [8]. They have shown the potential to effectively suppress tumors by enhancing the immune response [9]. However, the efficacy of immunotherapeutic approaches varies significantly due to the complex interactions of components within the tumor microenvironment (TME) [10].

The TME comprises various components, including not only tumor cells but also surrounding tissues, immune cells, blood vessels, and the extracellular matrix. They play significant roles in tumor growth, metastasis, and immune responses [11]. Understanding the diverse components of the TME is crucial for the development of novel treatment strategies, such as immune therapy [12].

Recently, single-cell RNA-seq technology has gained significant attention as an innovative tool for analyzing tumor cells and other components in the TME [13,14]. Using this technology, differences in gene expression within the TME can be precisely analyzed, allowing the exploration of new candidates for therapeutic targets and the development of novel treatment strategies [15]. Driven by recent advancements in the field, single-cell RNA-seq datasets for breast cancer tissues are being continuously generated and publicized.

In this retrospective study, we analyzed recently published single-cell RNA-seq datasets of breast cancer tissues to determine the composition of tumor cells and other components of the TME. We investigated differences in gene expression between tumor and normal tissues separately for each cell type and explored the interactions within the TME using a variety of analytical tools. The ultimate goal is to discover potential therapeutic targets that can be utilized for the treatment of patients with TNBC.

**Figure 1:**
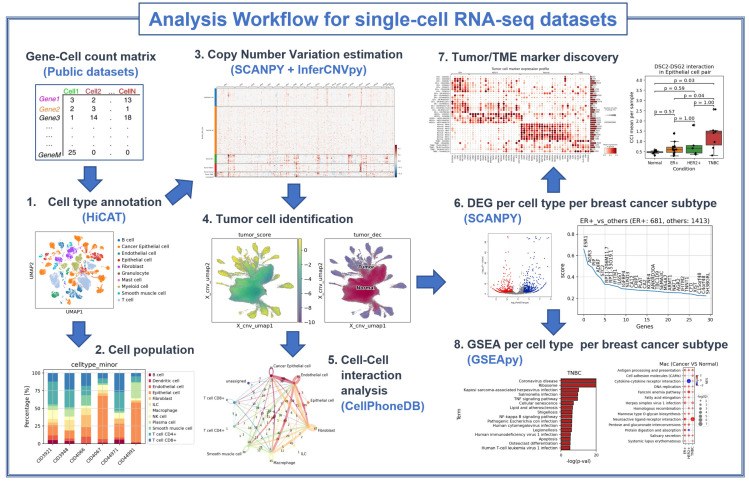
An illustration of the single-cell RNA-seq data analysis workflow. Within the RNA count matrices, the workflow starts with the annotation of cell types. An estimation of CNV was subsequently performed to identify tumor cells. Once cell types, including tumor cells, were identified, various analyses, such as differential gene expression (DEG), gene set enrichment analysis (GSEA), and inference of cell-to-cell interactions (CCI), were performed for each cell type. Subtype markers were also selected from the differentially expressed genes. This comprehensive workflow allowed us to explore the gene expression patterns per cell type and interactions within the tumor microenvironment, providing deeper insight into the behavior of tumor cells in vivo.

An illustration of the analysis workflow used in this study is depicted in Figure 1. Using publicly available datasets, we first used HiCAT [16] to annotate cell types within breast cancer tissues, in 3-level taxonomies, i.e., major type, minor type, and subset, and utilized InferCNVpy [https://github.com/icbi-lab/infercnvpy accessed on 1 May 2022] to identify tumor cells based on estimates of copy number variations (CNVs) for each cell. With these cell type annotation results, we used CellPhoneDB [17] to perform cell–cell interaction inference among different cell types and SCANPY [18] and GSEApy [19] to perform differentially expressed gene and gene set enrichment analysis. To obtain meaningful and unbiased results, random sampling was applied to ensure that the number of cells was roughly equal across all the samples. Using this workflow, we compared the three subtypes of breast cancer (HR-positive, HER2-positive, and TNBC) against normal tissue. 

There were several studies on TNBC using single-cell RNA-seq analysis, including the functional clustering of cancer-associated fibroblasts (CAFs) [20] and cell–cell interaction analysis for epithelial–mesenchymal transition characteristics [21]. In the former, CAFs in TNBC were grouped into three clusters, some of the markers of which were reported to have associations with patient survival. In the latter, TNBC cancer cells were classified into two groups, epithelium-like and mesenchymal-like, for which the cell–cell interaction analysis was performed to identify various therapeutic targets without experimentally validating their potentials. Compared to these works, more comprehensive analyses on TNBC were performed with different analytical procedures to be described later. We were able to identify novel markers and possible treatment targets for TNBC, some of which were not reported as potential targets of TNBC. The results were visualized to clearly distinguish the differences among subtypes. Specific marker genes for not only TNBC but also HR-positive and HER2-positive subtypes were identified. Markers for HR-positive and HER2-positive breast cancer were used to validate our workflow, while those for TNBC were used to explore the possible target genes and mechanisms of action that may be effective in treatment strategies for TNBC. This exploration focused particularly on gene-specific interactions and potential therapeutic targets within the context of TNBC.

## 2. Materials and Methods

### 2.1. Datasets Used

In this study, we used 3 public single-cell RNA-seq datasets, as summarized in Table 1. The cancer tissue data were obtained from surgical samples of breast cancer patients who underwent tumor resection surgery. The normal tissue data, on the other hand, came from surgical samples obtained through reduction mammoplasties in healthy individuals or prophylactic mastectomies in BRCA 1/2 mutation carriers. The cancer tissue data included clinical information categorized into subtypes. The GSE176078 [22] dataset consists of 12 HR-positive patients, 5 HER2-positive patients, and 9 TNBC patients. The GSE161529 [23] dataset contains 20 HR-positive patients, 6 HER2-positive, 8 TNBC, 7 lymph node metastasis patients, 4 preneoplastic, and 24 normal tissue samples; we did not use 7 lymph node sequencing data. GSE180878 [24] is composed of 4 reduction mammoplasties, 5 prophylactic mastectomies due to *BRCA1*/*2* mutations, and 7 normal tissue samples from the opposite side of breast cancer patients where cancer did not occur.

### 2.2. Cell Type Identification

For the three datasets below, cell type identification was performed first using HiCAT [16], a marker-based, hierarchical cell type annotation tool. Considering the heterogeneity of each sample, identification was conducted separately for each sample. For the marker database, we used the subset markers in Supplemental Table S1 obtained from R&D Systems (https://www.rndsystems.com/resources/cell-markers accessed on 1 March 2022). HiCAT annotates cell types in a 3-level taxonomy, i.e., it identifies major types, minor types, and subsets in a hierarchical way. We used major types to select the normal reference for InferCNVpy. We used minor types to perform cell–cell interaction, DEG, and gene set enrichment analysis.

### 2.3. Tumor Cell Identification

Tumor cell identification was performed using the CNV patterns obtained from the InferCNVpy package. We merged the three datasets into one and used the InferCNVpy package to estimate the CNV patterns for each cell. For reference cell types, we used all major cell types other than epithelial cells, i.e., endothelial, fibroblast, and immune cells. With the estimated CNV pattern and CNV score for each cell, we identified tumor cells as follows.

- Perform dimension reduction of CNV patterns using principal component analysis with 15 components to obtain PCA-transform CNV patterns.
- Use PCA-transform CNV patterns to construct neighbor graph with 15 neighbors per cell (kneighbors_graph method in the scikit-learn package).
- The neighbor graph was used to perform clustering via the Louvain algorithm with a resolution of 2. (We used the Louvain algorithm in the scikit-network package.)
- An aggregated adjacency matrix among clusters was obtained.
- The clusters for which the percentage of normal reference cells was greater than 20% were set as seed clusters. The other clusters are set “unprocessed”.
- We chose a cluster in the unprocessed pool. If its connectivity with the seed clusters was highest among others and greater than the minimum connectivity within the seed clusters, we added it to the seed clusters. This step is repeated until there are no unprocessed clusters that have connectivity higher than or equal to the threshold.
- Initial decision: Initially, those in the seed clusters were considered normal, while those in the unprocessed ones were considered tumors.
- With the initial decision, we obtained a Gaussian mixture model (GMM) using those marked as normal. (GaussianMixture method in the scikit-learn package).
- The negative GMM scores were then computed using the “score_samples” method to weigh the CNV scores obtained using InferCNVpy.
- A bimodal approximation of the weighted CNV scores was used to make the final decision.

### 2.4. Differentially Expressed Gene Analysis

Differentially expressed gene (DEG) lists were obtained separately for each cell type, including tumor cells (cancer epithelial cells), using Scanpy’s [18] rank_genes_groups method. To avoid sample bias due to high differences in the number of cells from each sample, we selected samples and cells from each sample as follows.

- For a specific cell type (target cell type), we first selected samples for which the number of cells of that type was larger than the lower cutoff for the number of target cells, e.g., 200.
- The upper cutoff for the number of cells was set to twice the minimum number of target cells among the selected samples.
- For samples with more target cells than the upper cutoff, we randomly selected cells up to the upper cutoff.

In A, the minimum number of cells per sample was set differently for each cell type since the cell populations were quite different from sample to sample. We tried to set the values such that not too many samples were excluded, while the number of total cells from all the selected samples was not too small. Using this procedure, we tried to ensure that the number of target cells was as high as possible while using as many cells as possible to obtain reproducible results. 

From the results of the rank_genes_groups method, we used the *p* values cutoff 0.05 to filter DEGs to perform the following gene set enrichment analysis. 

### 2.5. Selection of Marker Genes

As mentioned, we used the fraction of cells with nonzero expression in the target group and that in the reference group (i.e., pct_nz_group and pct_nz_reference). The marker gene score was defined as

Score = (nonzero fraction in target) × (1 − nonzero fraction in reference)

the same as the marker score in MarkerCount [25]. With this score and the two fractions, we chose M genes in descending order of the score if they met the following two conditions.

- nonzero fraction in target ≥ 0.5
- nonzero fraction in reference < 0.3

### 2.6. Gene Set Enrichment Analysis

The gene set enrichment analysis was performed using GSEApy [19] based on the differentially expressed gene lists obtained from the DEG results. Genes with a *p* value less than or equal to 0.05 and log2fold change greater than or equal to 0.25 were selected for GSEA. With these genes, the pre-rank method in GSEApy was used for enrichment analysis via the KEGG pathway database [26] (KEGG human 2021). The results were filtered to include only those with *p* values less than or equal to 0.05.

### 2.7. Cell-to-Cell Interaction Analysis

Cell–cell interaction analysis was subsequently conducted using the CellPhoneDB [17] tool. The analysis procedure was as follows.

- For each cell type, cell–cell interactions were inferred separately for each sample. We excluded samples that had fewer than 40 cells.
- We filtered out by the *p* value cutoff of 0.05 and mean cutoff of 0.1.
- For each subtype (condition), we collected the cell–cell interactions if they occurred in at least 75% of the samples under the same conditions.

## 3. Results

### 3.1. Cell Type Population Shows High Heterogeneity in Immune Cell Proportion Even within a Subtype, Indicating That Immune Reaction Is Not Subtype-Specific

As mentioned above, cell type annotation was performed individually for each dataset at three hierarchical levels: major type, minor type, and subset. The major cell types include epithelial cells, endothelial cells, fibroblasts, T cells, B cells, and myeloid cells. Further distinctions were made for immune cells to annotate their minor types, such as CD4+ T cells, CD8+ T cells, B cells, plasma cells, dendritic cells, and macrophages. The subsets were then further classified, i.e., CD4+ T cells were subdivided into Th1, Th2, Th9, Th17, Th22, and regulatory T cells, macrophages into M1, M2a, M2b, M2c, and M2d, and B cells into memory B cells, regulatory B cells, follicular B cells, and marginal zone B cells. The epithelial cells were further classified as either cancerous or normal by using the tumor cell identification procedure.

**Figure 2:**
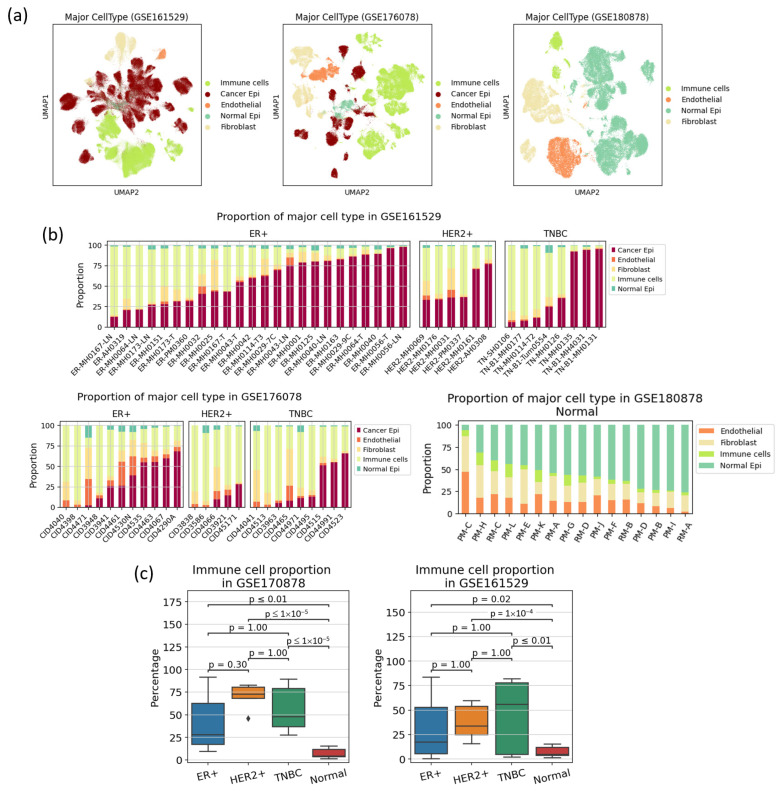
A summary of the cell type identification results. (**a**) UMAP plot of the major cell types in the three datasets, (**b**) bar plots representing the proportions of major cell types relative to the total number of cells per sample, and (**c**) box plots showing the distributions of immune cell proportions among breast cancer subtypes and normal tissue. For the TNBC and HER2-positive subtypes, there were samples with a high proportion of immune cells, probably corresponding to the so-called “hot tumors”.

It is evident from the UMAP plot that, unlike other normal cell types, cancer epithelial cells form various clusters, indicating heterogeneity across patients (Figure 2a). The proportion of each cell type relative to the total number of cells in each sample (Figure 2b) indicates that normal tissues consist predominantly of epithelial cells, while tumor tissues also have a large portion of immune cells. The proportions of cancer cells and immune cells varied significantly within each dataset of breast cancer samples, indicating the presence of different environments, referred to as “hot tumors” and “cold tumors”. Tumors are characterized by the activation of immune cells that can recognize and eliminate tumor cells [27,28]. The statistical significance in differences of immune cell populations among breast cancer subtypes were determined using *t* tests with Bonferroni correction (Figure 2c). Although some of the differences among the three subtypes were not as significant as those in normal tissue, the proportions of immune cells were found to be higher in HER2-positive and TNBC compared to HR-positive breast cancer. These findings showed consistent trends in both datasets. However, the immune cell population is shown to be highly heterogenous even within a subtype, indicating that immune reaction is not subtype-specific.

### 3.2. CNV-Based Tumor Cell Identification Results Also Show High Heterogeneity, Especially in the TNBC Subtype

**Figure 3:**
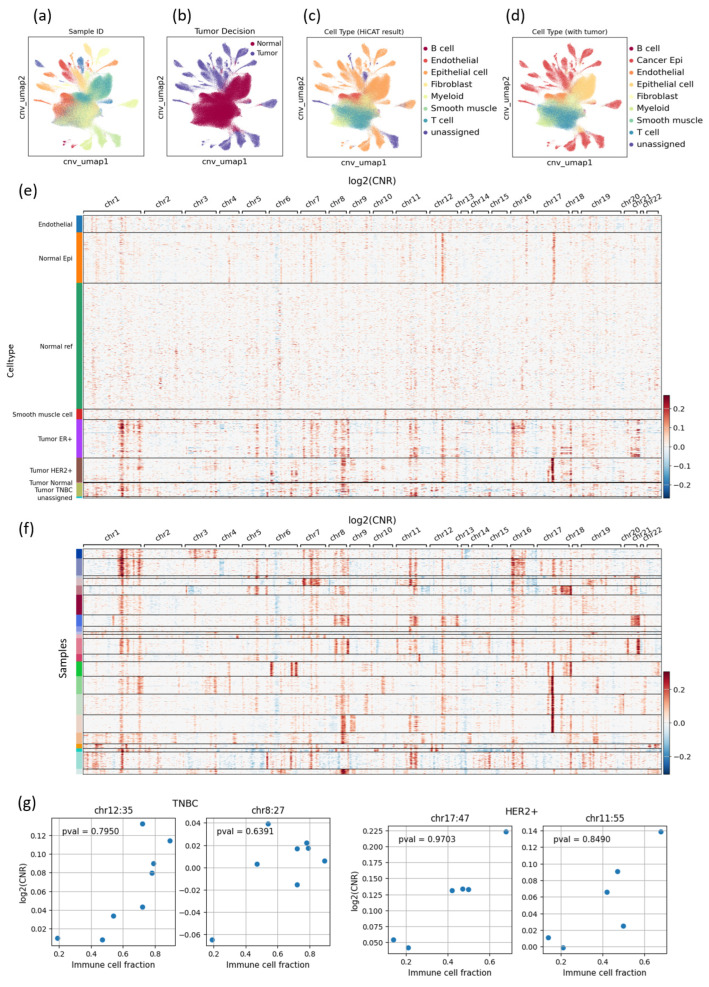
A summary of the estimated CNVs and UMAP projections. (**a**–**d**) illustrate the UMAP projections obtained using the estimated CNV patterns with different colors corresponding to: (**a**) sample ID, (**b**) tumor decision, (**c**) major cell type, and (**d**) the revised major cell type, including tumor cells (Cancer Epi). (**e**) Heatmap shows the estimated CNV patterns, in terms of log2(CNR), grouped according to cell type, where CNR is the copy number ratio, for which a positive value represents amplification, while a negative value means loss. The ‘Normal ref’ is the reference normal cell used to run InferCNVpy (including T cells, B cells, myeloid cells, and fibroblasts). ‘Normal Epi’ represents the normal epithelial cells from normal tissues or those detected as normal from tumor tissue. Tumor cells (Cancer Epi) were subdivided into tumor ER+, tumor HER2-positive, tumor TNBC, and tumor normal subsets, where ‘tumor normal’ is the cells classified as tumor but from normal tissue. However, the number of these cells was very small compared to that in tumor tissues. (**f**) It is the same heatmap showing only tumor cells grouped by sample ID, emphasizing the highly heterogenous nature of tumor cells. We showed only those samples having 400 or more tumor cells. (**g**) Correlation of log2(CNR) with the immune cell fraction in each sample. We took the average of log2(CNR)s on the tumor cells separately for each sample of a specific subtype. Then, we computed the Pearson correlation coefficient (PCC) with the immune cell fractions for each genomic spot where the CNR is computed. We could identify two spots for TNBC with PCC ≥ 0.6 and two for HER2-positive with 0.8.

The UMAP plots for the dimension-reduced CNV patterns of all cells in the merged dataset clearly show the high heterogeneity of tumor cells (Figure 3a–d). The clusters in the center mainly consist of normal cells, including immune cells, fibroblasts, and normal epithelial cells from normal tissues (Figure 3b,d). Despite being different cell types, they exhibit similar CNV patterns and appear adjacent to each other on the UMAP plot. On the other hand, the clusters in the outer regions are mostly mapped to epithelial cells from tumor tissues (cancer epithelial cells) and exhibit diverse cluster formations, clearly distinguishing each sample. This reflects the tumors’ heterogeneity in their CNV patterns. In contrast to the tumor cells in the outer clusters, the normal epithelial cells (from normal breast tissues) are centrally located, with similar CNV patterns across different samples, despite being from distinct individuals.

The comprehensive heatmap depicting the CNV patterns of all cells grouped according to their cell type shows the overall CNV landscape, emphasizing the subtype-specific CNV gain or loss (Figure 3e). The heatmap showing only tumor cells grouped according to their sample ID shows high heterogeneity across samples even within the same subtype (Figure 3f). Nevertheless, we could identify several commonalities across some samples or within the same subtype. Notably, the HER2-positive subtype exhibited high amplification on chromosome 17, consistent with the known fact that the HER2 gene resides on chromosome 17 and may undergo mutation and/or structural variation [29]. In some HR-positive samples, a common loss of chromosome 6 was observed, consistent with previous findings that alterations in several genes on chromosome 6 could influence the formation and development of breast cancer and other cancer types, such as ovarian cancer, melanoma, and leukemia [30]. Compared with other subtypes, tumor cells in the TNBC subtype exhibit distinct amplification of chromosomes 1 and 7, even if the amplification is not as high as that of chromosome 17 in the HER2-positive subtype. In summary, the CNV estimation results for each cell type appear to be reasonably accurate when considering the established knowledge, and scRNA-seq-based CNV analysis explains distinct gene alterations among different tumor subtypes. However, we see that they are highly heterogenous from sample to sample even within the same subtype, suggesting that there is room for improvement in therapeutic outcomes by applying personalized therapy in addition to subtype-specific strategies. 

In addition to its high heterogeneities of CNV patterns, we could also determine that the CNVs at some specific genomic spots had a high correlation with immune cell proportion (Figure 3g). Since the numbers of samples for TNBC and HER2-positive were limited, we cannot assess its statistical significance for now. However, it would be worth further investigation to check its biological mechanism.

### 3.3. Subtype-Specific Markers of Cancer Epithelial Cells Include Novel TNBC Markers

**Figure 4:**
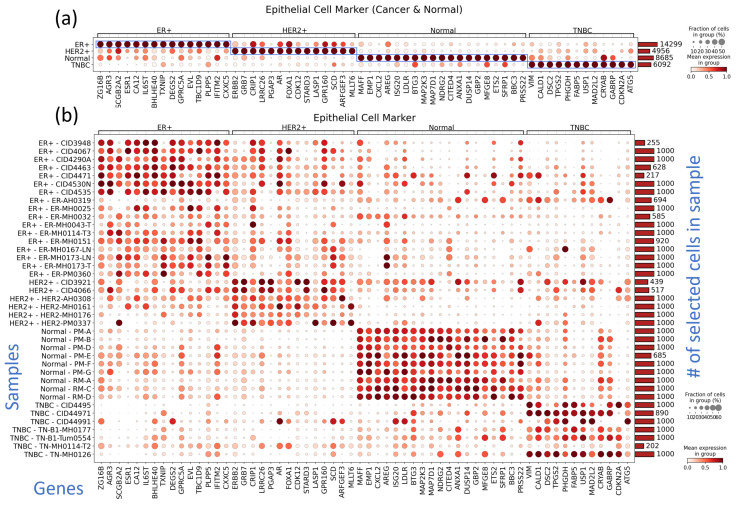
Dot plots showing the expression of subtype marker genes for epithelial cells grouped according to subtypes (**a**) and samples (**b**). For unbiased marker discovery, we selected cells from each sample such that the numbers of cells from all the samples are roughly equal. The samples having a number of epithelial cells smaller than the minimum value (in this case 250) were excluded from the figure. See Supplemental Figure S1 for other TME components, including macrophages, fibroblasts, endothelial cells, and CD8+ T cells. In (**a**), the blue boxes were added to highlight the subtype-specific markers we identified.

Even though the immune cell population and CNV patterns were highly heterogenous among samples even within the same subtype, we tried to identify common marker genes specific to a subtype, not only for tumor cells but also for other cell types in the TME, including endothelial cells, fibroblasts, macrophages, and CD8+ T cells. The results are summarized in Figure 4 for epithelial cells and Supplemental Figure S1 for macrophages, fibroblasts, endothelial cells, and CD8+ T cells. B cells and CD4+ T cells were excluded from the analysis because they were almost absent in normal tissues.

The marker discovery results (Figure 4a,b) clearly show the gene expression of the canonical markers of cancer epithelial cells for the HR-positive and HER2-positive subtypes, i.e., *ESR1* [31], *AGR2* [32] and *AGR3* [33] in the HR-positive subgroup and *ERBB2* [34] in the HER2-positive subgroup, indicating that the analysis is appropriate in this context. The per-sample marker expression pattern in (b) also shows specificities among subtypes, even though some marker genes for the HER2-positive subtype, e.g., *CRIP1*, *LRRC26*, *AR*, *FOXA1*, and *GPR160*, are also expressed in HR-positive samples. Notably, although AH0319 was classified as an HR-positive subtype, its expression pattern was more similar to that of TNBC, as its TNBC marker expression was greater than that of the HR-positive or HER2-positive subtypes. For the TNBC subtype, twelve marker genes were identified in the comparative analysis of TNBC cancer epithelial cells with those of normal and other subtypes. The twelve markers of TNBC cancer epithelial cell were summarized with their biological implication (Table 2).

The top twelve markers identified for TNBC cancer epithelial cells are genes known to have specific functions in tumor cells. TNBC cancer epithelial cells exhibit minimal expression of marker genes associated with other subtypes, suggesting that these cells are suitable candidates for use as marker genes for distinguishing TNBC from other breast cancer subtypes.

### 3.4. Markers of Other TME Components Show Clear Differences from Normal Tissue and Heterogeneity among Samples in Immune Cell Reaction 

In addition to cancer epithelial cells, we could also identify some markers of other TME components, including fibroblasts, macrophages, endothelial, cells and CD8+ T cells (Supplemental Figure S1). As shown, each breast cancer subtype exhibited distinct marker gene expression even though some of the markers were also expressed in some samples of other subtypes. However, when compared to samples of normal tissue, they display entirely distinct marker expression patterns for the same cell type. In particular, the TNBC subtype had markedly greater expression of these markers than did the other subtypes. This is especially the case for immune cells, such as CD8+ T cells and macrophages, for which the expression of these marker genes was very low in normal tissue. This observation highlights the unique and pronounced differences in gene expression patterns in various TME components between TNBC and other breast cancer subtypes and normal tissues. Although we could not identify distinct marker genes specific to a subtype for fibroblasts, endothelial cells, or macrophages in the HR-positive and HER2-positive subtypes, it is evident that the cell types constituting the TME in cancer patients exhibit entirely different gene expression patterns when compared to those in normal tissue.

### 3.5. Cell–Cell Interactions Analysis Identified Novel TNBC-Specific Interactions Including DSC2-DSG2, Suggesting That TNBC Tumor Cells Are More Tightly Aggregated 

**Figure 5:**
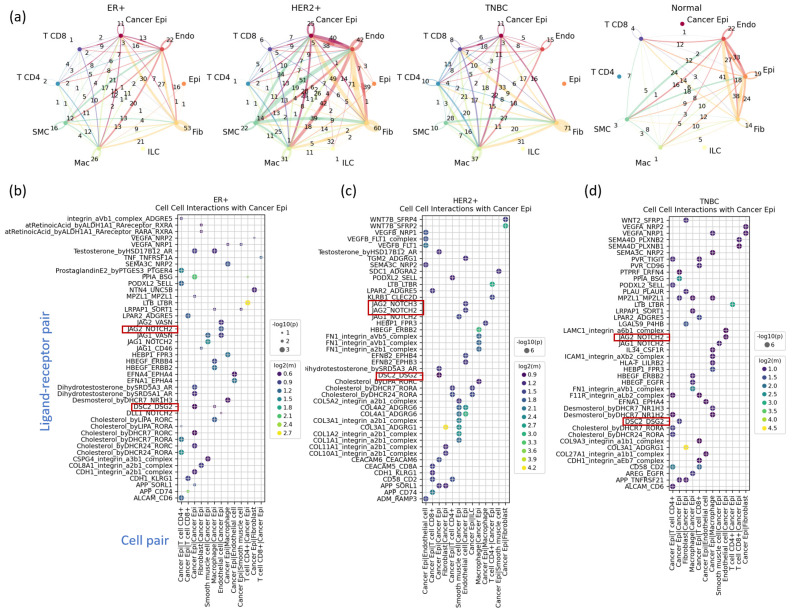
A summary of the cell–cell interaction analysis. In (**a**), the line width corresponds to the number of inferred interactions (the numbers on the lines) between a pair of cell types, including cancer epithelial cells. (**b**–**d**): Three dot plots are the cell–cell interactions found in each subtype between cancer epithelial cells and with other cell types. For each subtype, the identified interactions were first filtered by *p* value ≤ 0.05 and mean ≥ 0.5. The filtered interactions were then sorted first by *p* values and then by mean value if *p* values are equal, i.e., the same to zero. Finally, fifty highest interactions were taken according to the sorted order. In (**b**–**d**), we highlighted the two interactions, *JAG2-NOTCH2* and *DSC2-DSG2*, that should be noted.

The cell–cell interaction analysis results show how cancer epithelial cells interact with the main players in the tumor microenvironment, including fibroblasts, macrophages, endothelial cells, CD8+ T cells, CD4+ T cells, and tumor cells themselves (cancer epithelial cells) (Figure 5a). Among many interactions specific to each subtype, we noticed *JAG-NOTCH* and *DSC2-DSG2* interactions (Figure 5b–d).

The *JAG-NOTCH* interaction between tumor cells and endothelial cells occurred in all subtypes. The *JAG-NOTCH* interaction in breast cancer has several biological implications; i.e., it can drive the transformation of normal breast cells into cancer cells. Notch signaling promotes uncontrolled cell growth and proliferation. It maintains cancer stem cells, which contribute to tumor recurrence. It also supports the formation of blood vessels within tumors and may help cancer cells evade the immune system. Targeting this pathway is being explored for breast cancer treatment.

**Figure 6:**
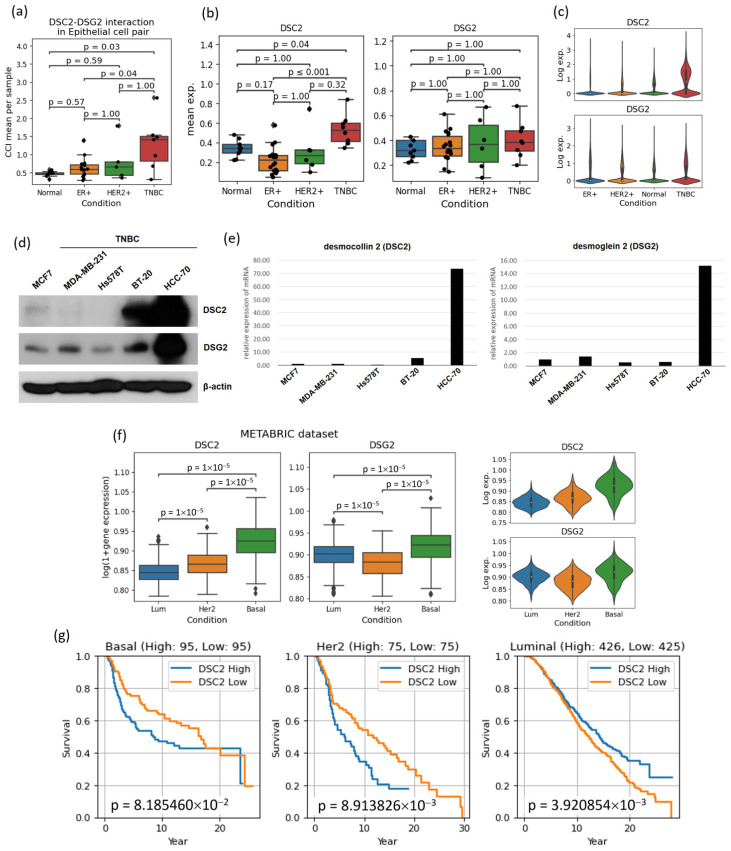
A summary of verification of *DSC2-DSG2* interaction and their gene expressions. (**a**): The box plot shows *DSC2-DSG* interaction is significantly higher in TNBC than others. (**b**,**c**): The boxplot and the violin plot on the *DSC2* and *DSG2* gene expression confirms the stronger interaction of *DSC2-DSG2* in TNBC. (**d**,**e**): Protein and mRNA level measurements using Western blotting and RT-PCR on one HR-positive breast cancer cell line (MCF7) and four TNBC cell lines (MDA-MB-231, Hs578T, BT-20, and HCC-70) showed also increased mRNA and protein levels of *DSC2* and *DSG2*. The uncropped original Western blots were shown in Supplemental Figure S2. (**f**): The *DSC2* and *DSG2* gene expression differences across subtype could also be identified in the METABRIC dataset. (**g**): Survival comparison of patients with high and low *DSC2* expression shows it prognostic power in all three subtypes. For *DSC2* high and low, we stratified patients into three groups of equal number according to the sorted order of *DSC2* expression level and compared high and low groups, excluding the middle group.

Another notable interaction was the interaction between *DSC2* and *DSG2* in tumor cells, where the former is one of the 12 TNBC markers we identified. Although these interactions were detected in all of the subtypes, they had different levels of interaction: strongest in TNBC, moderate in HER2-positive, and weakest in HR-positive patients (Figure 6a–c). Statistical tests on the per-sample interaction strength showed that the ones in TNBC were significantly higher than those in other subtypes (Figure 6a). The expression levels of these genes in different subtypes also support this argument (Figure 6e,f). 

The DSC and DSG gene families mediate cell adhesion functions through interactions in the intercellular space [47]. This cell adhesion structure is crucial for maintaining the normal architecture of epithelial tissues. While *DSC2* is involved in tumor progression and development in various types of cancer, it is known to be highly expressed in aggressive subtypes and to be correlated with poor survival outcome in patients with breast cancer [48]. Additionally, a recent experimental study of a breast cancer cell line revealed significantly greater expression of the *DSC2* gene in HER2-positive patients and TNBC patients, and *DSC2* overexpression was significantly correlated with shorter disease-free survival and overall survival. Furthermore, tumor cells with high *DSC2* expression exhibit increased metastatic potential, tumor cell cohesion, and resistance to chemotherapy [37].

Inspired by the results and the previous studies, we further verified the mRNA and protein levels of *DSC2* and *DSG2* in one HR-positive cell line and four TNBC cell lines, using quantitative real-time polymerase chain reaction (RT-PCR) and western blotting (Figure 6d,e). As expected, there was a notable increase in both the mRNA and protein levels of *DSC2* and *DSG2* in some TNBC cell lines. Verification using the METABRIC dataset [49] showed not only a similar tendency in its gene expression differences across subtype, but also its power as a prognostic marker for all three subtypes (Figure 6f,g). Of note, while high *DSC2* levels showed worse survival in TNBC and HER2-positive breast cancer, it was shown to be reversed in HR-positive breast cancer.

### 3.6. Gene Set Enrichment Analysis Shows Subtype-Specific Cell State Changes in Various TME Components

**Figure 7:**
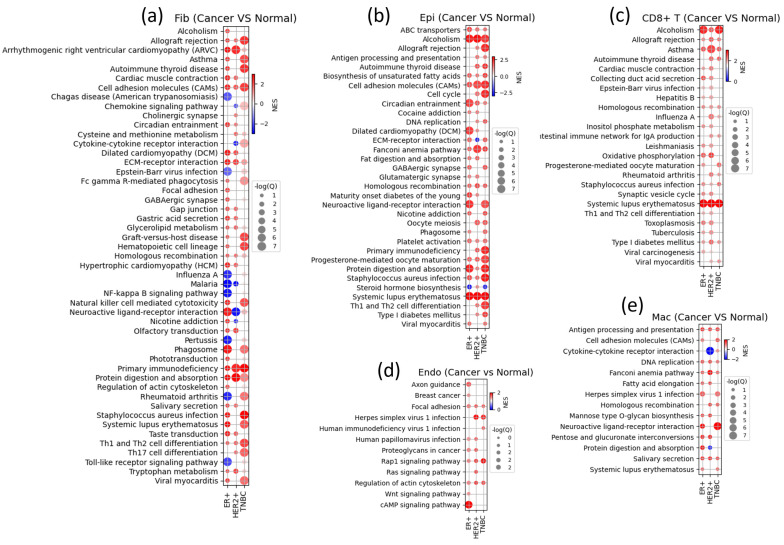
A summary of the results of the gene set enrichment analysis for (**a**) fibroblasts, (**b**) epithelial cells, (**c**) CD8+ T cells, (**d**) endothelial cells, and (**e**) macrophages. The figures show the upregulated (in red) and downregulated (in blue) pathways in each breast cancer subtype. The dot size indicates the significance of the results proportional to −log_10_ (*p* value).

The gene set enrichment analysis results provide us an insight into the cell state of each TME component (Figure 7). For each cell type, we used subtype-specific DEGs, including the marker genes shown in Figure 4 and Supplemental Figure S1. In TNBC, epithelial cells exhibit increased activation of several pathways, including those related to allograft rejection, the cell cycle, DNA replication, primary immunodeficiency, and Th1/Th2 cell differentiation. The results suggest that TNBC cells undergo rapid cell cycle progression and that various immune regulation processes are more prominently activated (even if not all as shown in cell type population). 

Fibroblasts are involved in allograft rejection, autoimmune thyroid disease, cell adhesion molecules, graft-versus-host disease, hematopoietic cell lineage, natural killer cell-mediated cytotoxicity, Th1 and Th2 cell differentiation, and Th17 cell differentiation, suggesting that fibroblasts are involved not only in immune regulation-related pathways but also in processes related to cell adhesion, hematopoietic cell development, and potential roles in cellular transitions. 

Macrophages were shown to be active in the neuroactive ligand–receptor interaction pathway, while endothelial cells were active in human immunodeficiency virus 1 infection and the RAP1 signaling pathway. These findings indicate the involvement of specific cellular interactions and responses in these cell types, possibly related to immune responses or other functions associated with these pathways. The CD8+ T cells in the TNBC subtype exhibited upregulated expression in association with the HR-positive or HER2-positive subtypes.

## 4. Discussion

The results of this work clearly show that the identified cell types, including epithelial cells, endothelial cells, fibroblasts, and immune cells, in normal tissue and in the three breast cancer subtypes exhibited distinct gene expression patterns. Although there exist high heterogeneities even within a single subtype, we could identify subtype-specific marker genes of various TME components and several novel interactions between them. Even with these heterogeneities, there was an increased population of immune cells in cancer tissues, especially in HER2-positive and TNBC. These findings were consistent with previous studies. Li et al. reported the analysis of the TME based on breast cancer subtypes using the multi-omics datasets of breast cancer from the METABRIC cohort [49]. Basal-like and HER2-positive subtypes showed an association with a high immune score, while luminal subtypes were associated with a low immune score. Many studies also support the statement that TNBC is the most immunogenic among breast cancer, as evidenced by high tumor infiltrating lymphocytes in the TME [50]. Compared to other subtypes, TNBC exhibits higher significance and enrichment scores in pathways related not only to the immune response but also to cell cycle, DNA replication, and cell adhesion molecules. Another novel finding was the possible correlation of immune cell population with CNVs at several genomic spots, specifically in TNBC and HER2-positive subtypes, though it requires further investigation.

In the cell–cell interactions analysis, we found *JAG-NOTCH* interactions between tumor cells and endothelial cells. The Notch pathway is involved in tumor progression through various mechanisms such as stem cell maintenance, cell proliferation, and migration/invasion [51,52]. These studies reported that the Notch pathway regulates many components of the TME, including immune cells, fibroblast, endothelial, and mesenchymal cells. Specifically, Notch signaling in endothelial cells can contribute to tumor angiogenesis and play a critical role in the regulation of TME plasticity by promoting the endothelial-to-mesenchymal transition. Further research is needed to explore whether the Notch signaling in endothelial cells could be a promising therapeutic target in breast cancer.

Furthermore, we identified the top twelve marker genes of TNBC epithelial cells, namely, *VIM*, *CALD1*, *DSC2*, *TPGS2*, *PHGDH*, *FABP5*, *USP1*, *MAD2L2*, *CRYAB*, *GABRP*, *CDKN2A*, and *ATG5*. Some of these genes have been proven in previous studies to play important roles in various functions related to tumor cell formation, growth, and metastasis. Among these genes, we focused on the higher expression of the *DSC2* gene and the increased interaction between *DSC2* and *DSG2* in the TNBC subtype.

*DSC2* and *DSG2* are known to play key roles in desmosomes, which are cell junctions that mediate cell–cell adhesions to maintain tissue integrity [53]. In desmosomes, the transmembrane proteins desmocollin (*DSC1*-*3*) and desmoglein (*DSG1*-*4*) are intracellularly connected to the intermediate filament cytoskeleton through desmosomal plaque proteins. All desmosomes contain at least one DSC and one DSG, and both are required for adhesion [53,54]. In addition to their critical roles in adhesion, desmosomal proteins are emerging as mediators of cell signaling that is important for proper cell and tissue function [53,54]. There is a growing body of evidence for the roles of desmosomes in different types of cancer [53,54]. The expression of various desmosomal proteins has shown both tumor-suppressive and tumor-promoting effects on growth and metastasis in various types of cancer. Alterations in the expression of desmosomal components could promote cancer cell growth by modifying various intracellular signal transduction pathways [55]. Desmosomes are also involved in intercellular adhesion, resulting in cell aggregation and epithelial–mesenchymal transition [55]. *DSG2* has been associated with a poor prognosis and increased recurrence risk in breast cancer patients and is linked to an increased metastatic potential of breast cancer cells by promoting cell clustering and enhancing cell survival during tumor cell dissemination [56]. Recently, Reimer et al. reported that high *DSC2* expression was linked to a higher tumor grade and shorter disease-free survival in patients with TNBC. Moreover, the use of *DSC2* expression as a predictive marker for the development of brain and lung metastasis is interesting [37]. Consistent with the findings of previous studies, this analysis confirmed the specific expression of *DSC2* in TNBC tumor cells. Furthermore, cell–cell interaction analysis revealed that the *DSC2* gene interacts with the *DSG2* gene within tumor cells, and pathway analysis verified the upregulation of the *DSC2–DSG2* protein complex structure specifically in TNBC. To date, most studies have focused on the role of a single desmosomal protein in cancer. However, our results suggest that the interaction between *DSC2* and *DSG2* may be more important in the pathogenesis of TNBC, as may the expression of *DSC2* itself. However, further studies are needed to characterize the functions of the *DSC2-DSG2* complex in the regulation of cancer progression and metastasis.

Considering the roles of the *DSC2-DSG2* complex in TNBC reported in these studies, these complexes may be potential therapeutic targets for new drug discovery. Recently, antibody–drug conjugates (ADCs) that bind antibodies and cytotoxic drugs with a linker have led to a new era of targeted cancer therapy. One of the most important aspects of ADC development is the identification of the unique antigenic target of the monoclonal antibody component. The requirements for good antigen candidates are high expression in tumors, low expression in normal cells, and surface displacement available to the circulated monoclonal antibody [37,57]. Enfortumab vedotine, which targets the cell adhesion molecule Nectin-4, was approved for the treatment of advanced urothelial carcinoma [58]. The success of enfortumab vedotine in targeting cell adhesion molecules such as *DSC2* empowers the possibility of *DSC2* as a new drug target. Thus, further research is warranted to investigate the potential of the *DSC2-DSG2* complex as a new antigenic target in patients with advanced TNBC.

In addition, inhibiting the *DSC2* gene or weakening the desmosome protein structure formed by binding to the *DSG2* gene could weaken the strong cell–cell adhesion observed in TNBC. This approach may enhance the efficacy of chemotherapy and overcome resistance, particularly in TNBC, while also offering the potential to prevent metastasis by inhibiting further tumor cell adhesion.

Among the other cell types, fibroblasts in TNBC exhibit an overall upregulation of the expression of genes involved in the NF kappa B signaling pathway. The genes regulated through this pathway include those involved in the formation of protein complexes related to cell survival, such as Bcl-xl, and are upregulated in the expression of genes associated with cell cycle regulation in response to DNA damage, such as those associated with the ATM and NEMO protein complexes. Consequently, CAFs in TNBC cells evade cell death through cell cycle regulation in response to DNA damage, suggesting a potential therapeutic target for TNBC. In recent years, many studies have been conducted on the effect of CAFs on tumor initiation, progression, and therapeutic resistance in various types of cancer, including breast cancer [59,60,61]. However, there are still many challenges in understanding the precise roles of CAFs in each subtype of breast cancer. Our findings support the potential of CAFs as novel and promising therapeutic targets in TNBC and the need for further investigations.

## 5. Conclusions

In this work, not only did we confirm previous findings, but our rigorous data mining of published single-cell RNA-seq datasets also enabled us to identify previously unnoticed or overlooked features of TNBC. These studies might contribute to the discovery of specific biomarkers of TNBC, corresponding targeted treatments, and the exploration of the interaction mechanisms between tumor cells and other components of the TME.

## Figures and Tables

**Table 1:** Data summary.

	Tissue Type	Num. Cells	Num. Genes	Num. Samples
GSE176078	Tumor	100,064	29,733	26 (12, 5, 9, 0, 0)
GSE161529	Tumor/Normal	428,024	33,538	62 (20, 6, 8, 4, 24)
GSE180878	Normal	52,681	20,437	16 (0, 0, 0, 0, 16)

The numbers in parenthesis are the number of samples for ER+, HER2+, TNBC, Preneoplastic and Normal, respectively; In GSE161529, we did not consider 7 lymph node sequencing samples from the same ER+ patients.

**Table 2:** A summary of identified markers for TNBC cancer epithelial cells.

Gene	Description	Reference
*VIM*	Treatment-induced upregulation of *VIM* is associated with increased invasion in breast cancer cells; silencing it reduces metastasis, suggesting therapeutic promise.	[35]
*CALD1*	Upregulated *CALD1* gene expression in breast cancer is linked to aggressive tumor traits, such as heightened growth rate and cell motility, fostering carcinogenesis via epithelial–mesenchymal transition induction.	[36]
*DSC2*	In epithelial cells, *DSC2* encodes desmocollin protein, which is closely linked to heart disease and plays a crucial role in cell adhesion. However, its overexpression in tumors increases cell cohesion, hindering chemotherapy diffusion and promoting tumor growth. This is particularly associated with poor prognosis in aggressive subtypes like HER2-positive and TNBC.	[37]
*TPGS2*	*TPGS2* is pivotal in modulating the tumor microenvironment (TME) and fostering metastasis in breast cancer via the circ-*TPGS2*-mediated miR-7/*TRAF6*/NF-κB signaling cascade, creating a feedback loop that enhances the pro-metastatic impact of circ-*TPGS2*.	[38]
*PHGDH*	Overexpression of *PHGDH* enzyme is a hallmark of cancer cells, linked to accelerated cell division and proliferation, with ongoing research aiming to develop targeted therapies; in TNBC, it tends to be upregulated, often associated with CK5-positive cell lineage across diverse tissues and cancer types.	[39]
*FABP5*	Within breast tissue, *FABP5* facilitates fatty acid delivery, supporting cancer cell proliferation; its upregulation in prostate and breast cancer cells, notably in HR-negative breast cancer with poor prognosis, correlates with EpCAM upregulation, driven by c-Myc to promote tumor cell survival and progression.	[40]
*USP1*	Mutations in *USP1* can disrupt DNA repair regulation, potentially leading to cancer development; meanwhile, the *USP1*/*WDR48* complex mediates TGF-β signaling, enhancing TNBC cell mobility and EMT.	[41]
*MAD2L2*	Dysregulation or mutation of *MAD2L2*, involved in error detection and regulation of cell division, impacts tumor development and growth, drawing attention as a potential therapeutic target. Particularly, its overexpression in MDA-MB-157 TNBC cells correlates with enhanced cell division promotion compared to other breast cancer subtypes.	[42]
*CRYAB*	Highly expressed in several cancer types, including breast, cervical, and renal cell carcinoma, *CRYAB* binds to proteins, promoting cell survival and exerting a protective role. In breast cancer, it is associated with tumor invasion, metastasis, and prognosis, with breast epithelial cells expressing elevated *CRYAB* levels showing tumor-like growth patterns in vitro, implicating *CRYAB* in tumor formation.	[43]
*GABRP*	Recent research confirms activation of the *EGFR* signaling pathway in TNBC, where abundant *GABRP* in TNBC stem cells interacts with *EGFR*, sustaining its high expression levels and elevating chemoresistance. Inhibiting *GABRP*-induced *EGFR* signaling, which includes paclitaxel, doxorubicin, and cisplatin, holds promise for improving chemotherapy response.	[44]
*CDKN2A*	In a recent study of 587 TNBC patients, tumor samples were categorized into six subtypes, revealing abnormal amplification of the *CDKN2A*/*B* gene in the basal-like 1 type. Our findings indicate upregulated *CDKN2A* expression in TNBC patients, possibly due to defects in the *CDKN2A* gene itself, known as a tumor suppressor that halts the cell cycle during the G1 phase.	[6,45]
*ATG5*	*ATG5* and *ATG2B* are regulated by miR-181a, which weakens cancer stem cells and enhances autophagy in TNBC. This suggests *ATG5′*s role in suppressing cancer stem cells in TNBC, offering potential for TNBC treatment using compounds like curcumin.	[46]

## Data Availability

All the single-cell RNA-seq data are available from the Gene Expression Omnibus (GEO, https://www.ncbi.nlm.nih.gov/geo/ accessed on 1 June 2022). The annotated results in Anndata-formatted h5ad file were deposited in the Figshare (https://figshare.com/articles/dataset/Annotated_single-cell_RNA-seq_dataset_used_in_A_Retrospective_View_on_Triple_Negative_Breast_Cancer_Microenvironment_Novel_Markers_Interactions_and_Mechanisms_of_Tumor-Associated_Components_using_public_Single-cell_RNA_Seq_Datasets_/24678549 accessed on 1 June 2022).

## Supplementary Materials

The following supporting information can be downloaded at: https://www.mdpi.com/article/10.3390/cancers16061173/s1, Figure S1: Dot plots showing the expression of subtype marker genes for other TME components than cancer epithelial cells, including macrophages, fibroblasts, endothelial cells, and CD8+ T cells; Figure S2: The uncropped original Western blots of the one in Figure 6d; Table S1: Cell-type markers used.
