# Supplementary material for: A Retrospective View of the Triple-Negative Breast Cancer Microenvironment: Novel Markers, Interactions, and Mechanisms of Tumor-Associated Components Using Public Single-Cell RNA-Seq Datasets
Source: Cancers (Basel). 2024 Mar 16;16(6):1173. doi: 10.3390/cancers16061173 (PMC10969223; doi:10.3390/cancers16061173)
Supplement: Supplementary file 1 [file cancers-16-01173-s001.zip › cancers-2876407-supplementary.pdf]

Supplemental Figures & Tables

# A Retrospective View of the Triple-Negative Breast Cancer Microenvironment: Novel Markers, Interactions, and Mechanisms of Tumor-Associated Components using Public Single-cell RNA-seq Datasets

Minsoo Kim[1], Wonhee Yang[2], Dawon Hong[3], Hye Sung Won[4†], Seokhyun Yoon[5†]

(a)

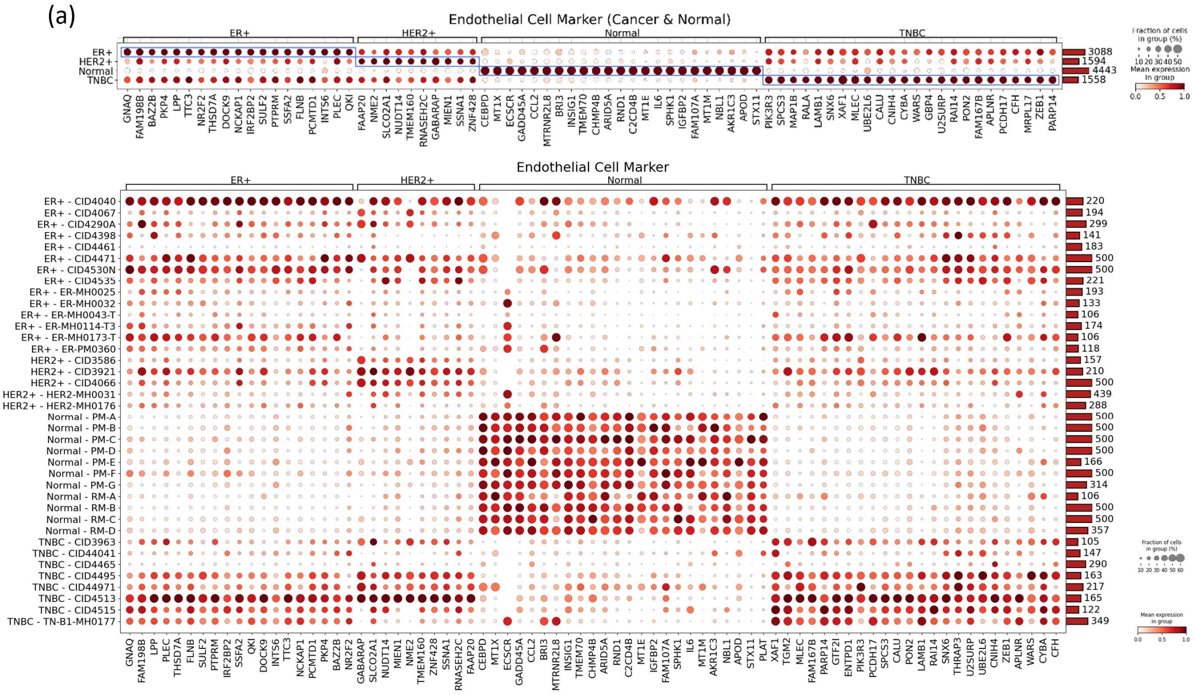


(b)

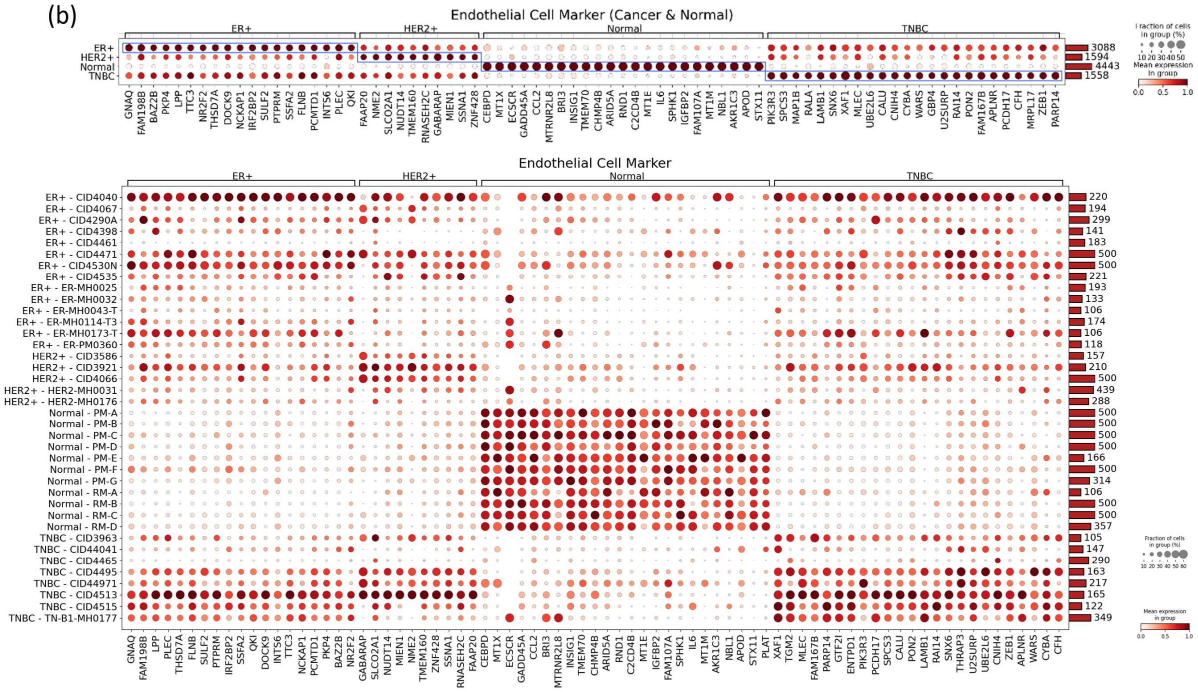

(c)

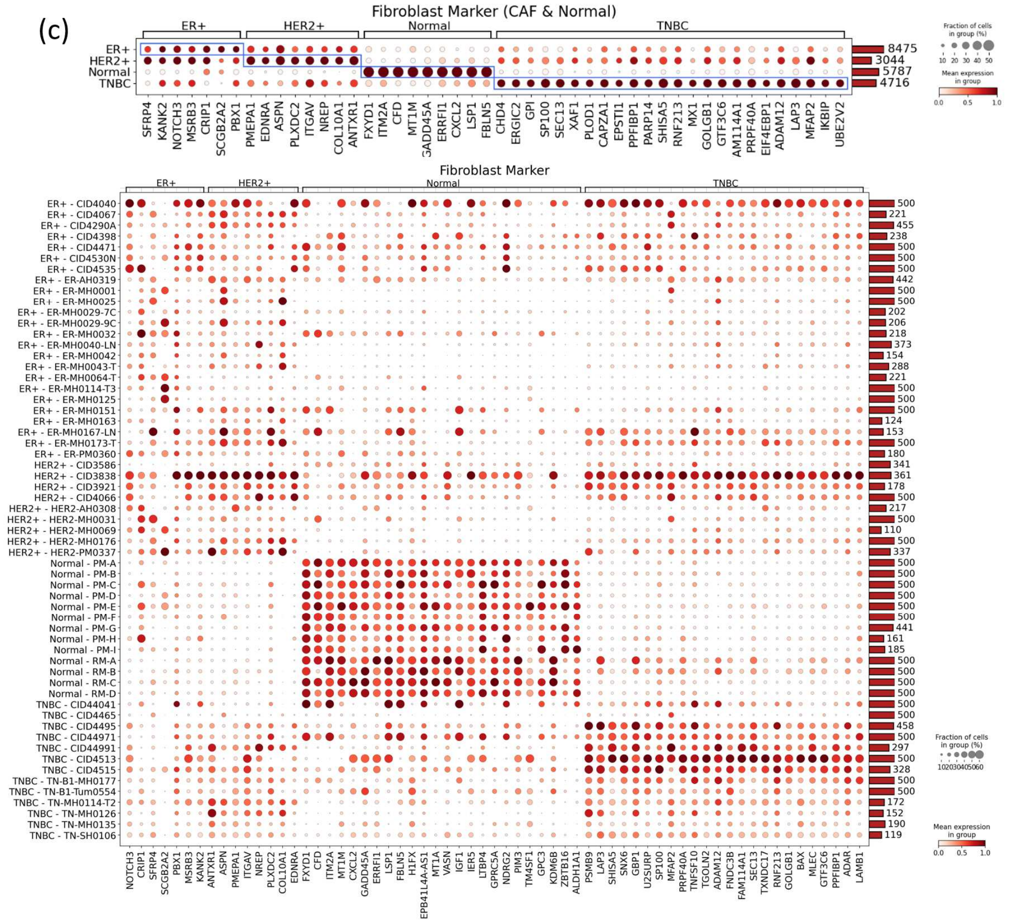


(d)

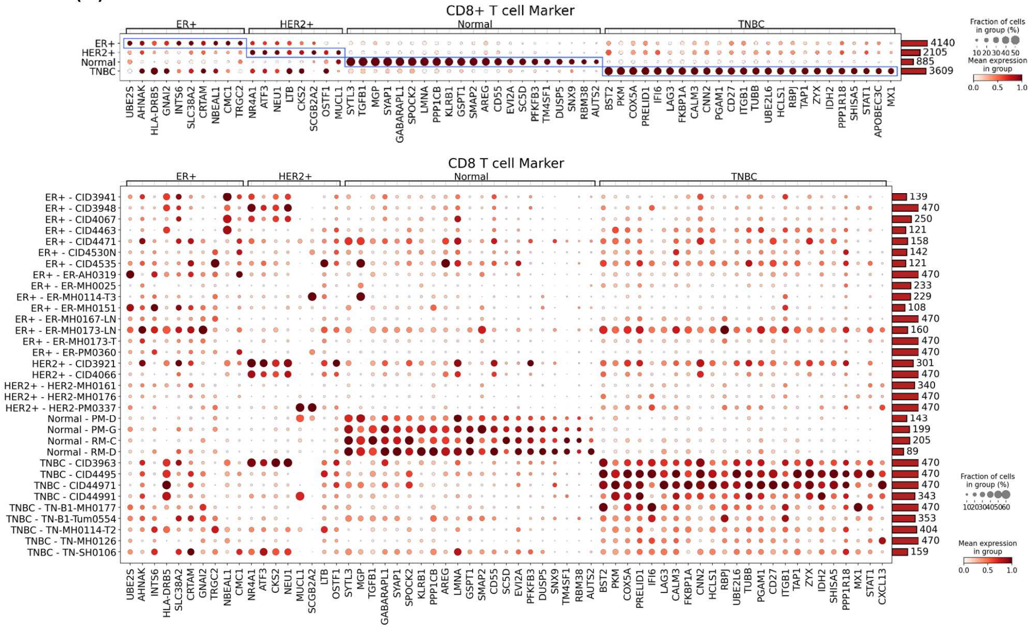


**Supplemental Figure S1** Dot plots showing the expression of subtype marker genes for other TME components than cancer epithelial cells, including macrophages, fibroblasts, endothelial cells, and CD8+ T cells. For each celltype, the marker expression was grouped according to subtype (top) and sample (bottom). For unbiased marker discovery, we selected cells from each sample such that the numbers of cells from all the samples are roughly equal. The samples having the number of cells smaller than the minimum value (in this case 250) were excluded from the figure.

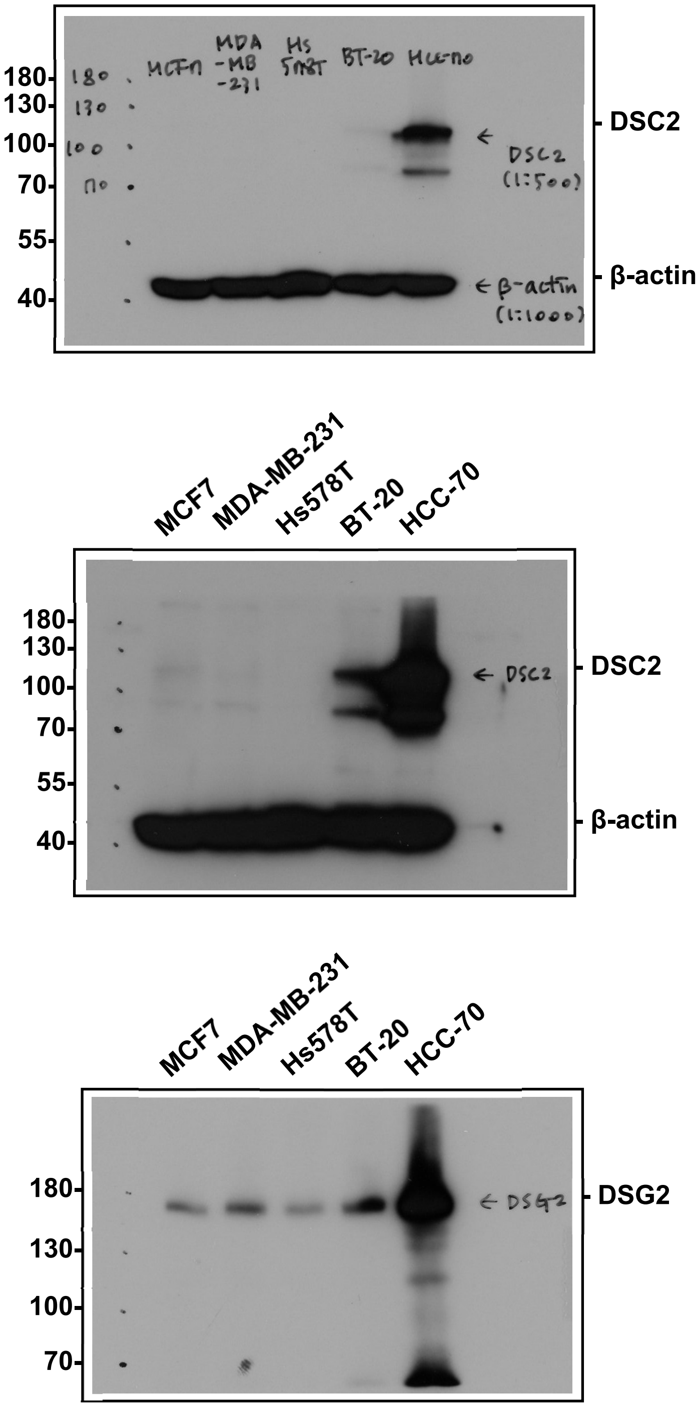


**Supplemental Figure S2** The uncropped original Western blots of the one in Figure 6d.

Supplemental Table S1. Cell-type markers used. They were mostly obtained from R&D systems, https://www.rndsystems.com/resources/cell-markers

| tissue | cell_type_major | cell_type_minor | cell_type_subset | exp | markers |
|---|---|---|---|---|---|
| Immune | T cell | T cell CD4+ | T cell (Tfh) | pos | CD3D,CD3E,CD3G,CD4,B3GAT1,BCL6,BTLA,CD40LG,CD84,CXCR4,CXCR5,ICOS,IL6R,MAF,MME,PDCD1,SLAMF1,STAT3,TNFRSF4 |
| Immune | T cell | T cell CD4+ | T cell (Tfh) | neg | CD14,CD19,CD8A,CD8B,SELL |
| Immune | T cell | T cell CD4+ | T cell (Tfh) | sec | CXCL13,IFNG,IL10,IL17A,IL17F,IL21,IL4 |
| Immune | T cell | T cell CD4+ | T cell (Th1) | pos | CD3D,CD3E,CD3G,CD4,CCR1,CCR5,CXCR3,IFNGR1,IFNGR2,IL12RB2,IL18R1,IL27RA,STAT1,STAT4,TBX21 |
| Immune | T cell | T cell CD4+ | T cell (Th1) | neg | CD14,CD19,CD8A,CD8B,SELL |
| Immune | T cell | T cell CD4+ | T cell (Th1) | sec | IFNG,IL2,LTA,TNF |
| Immune | T cell | T cell CD4+ | T cell (Th2) | pos | CD3D,CD3E,CD3G,CD4,CCR3,CCR4,CCR8,CRLF2,CXCR4,GATA3,IL17RB,IL4R,IRF4,IL1RL1,STAT5A,STAT5B,STAT6 |
| Immune | T cell | T cell CD4+ | T cell (Th2) | neg | CD14,CD19,CD8A,CD8B,SELL |
| Immune | T cell | T cell CD4+ | T cell (Th2) | sec | IL4,IL5,IL9,IL10,IL13,IL21 |
| Immune | T cell | T cell CD4+ | T cell (Th9) | pos | CD3D,CD3E,CD3G,CD4,IL17RB,IL4R,IRF4,SPI1,TGFBR2 |
| Immune | T cell | T cell CD4+ | T cell (Th9) | neg | CD14,CD19,CD8A,CD8B,SELL |
| Immune | T cell | T cell CD4+ | T cell (Th9) | sec | CCL17,CCL22,IL9 |
| Immune | T cell | T cell CD4+ | T cell (Th17) | pos | CD3D,CD3E,CD3G,CD4,BATF,CCR4,CCR6,IL1R1,IL21R,IL23R,IL6R,IRF4,RORA,RORC,STAT3,TGFBR2 |
| Immune | T cell | T cell CD4+ | T cell (Th17) | neg | CD14,CD19,CD8A,CD8B,SELL |
| Immune | T cell | T cell CD4+ | T cell (Th17) | sec | CCL20,IL17A,IL17F,IL21,IL22,IL26 |
| Immune | T cell | T cell CD4+ | T cell (Th22) | pos | CD3D,CD3E,CD3G,CD4,AHR,BATF,CCR10,CCR4,CCR6,IL6R,STAT3,TGFBR2,TNFRSF1A |
| Immune | T cell | T cell CD4+ | T cell (Th22) | neg | CD14,CD19,CD8A,CD8B,SELL,KLRB1 |
| Immune | T cell | T cell CD4+ | T cell (Th22) | sec | CCL7,CCL15,FGF1,FGF2,FGF3,FGF4,FGF5,FGF6,FGF7,FGF8,FGF9 |
| Immune | T cell | T cell CD4+ | T cell (Treg) | pos | CD3D,CD3E,CD3G,CD4,CD5,CTLA4,ENTPD1,FOXP3,IKZF2,IL10,IL2RA,ITGAE,IZUMO1R,LAG3,LAP3,LGALS1,LRRC32,NRP1,NT5E,SELL,STAT5A,STAT5B,TGFB1,TGFB2,TGFB3,TNFRSF4,TNFRSF18 |
| Immune | T cell | T cell CD4+ | T cell (Treg) | neg | CD14,CD19,CD8A,CD8B |
| Immune | T cell | T cell CD4+ | T cell (Treg) | sec | IL10,LGALS1,TGFB1,TGFB2,TGFB3,EBI3,IL12A |
| Immune | T cell | T cell CD4+ | T cell (Naive) | pos | CD3D,CD3E,CD3G,CD4,CCR7,SELL,IL7R,LEF1 |
| Immune | T cell | T cell CD4+ | T cell (Naive) | neg | CD14,CD19,CD8A,CD8B,IL2RA,KLRG1 |
| Immune | T cell | T cell CD8+ | T cell (Cytotoxic) | pos | CD3D,CD3E,CD3G,CD8A,CD8B,ZNF683,LAG3,ZFP36 |
| Immune | T cell | T cell CD8+ | T cell (Cytotoxic) | sec | IFNG,GZMB,GZMK |
| Immune | T cell | T cell CD8+ | T cell (Cytotoxic) | neg | CD14,CD19,CD4 |
| Immune | T cell | ILC | ILC1 | pos | IL2RA,PTPRC,ITGA1,CD69,IL2RB,IL7R,KLRB1,CXCR3,ICOS,IL1R1,IL12RB2,NCR1,TBX21 |
| Immune | T cell | ILC | ILC1 | neg | PTGDR2,IL17RB,IL23R,NCAM1,NCR2,IL1RL1,EOMES |
| Immune | T cell | ILC | ILC1 | sec | IFNG,TNF,IL2,LTA |
| Immune | T cell | ILC | ILC2 | pos | IL2RA,PTPRC,THY1,IL7R,KLRB1,PTGDR2,ICOS,IL1R1,IL17RB,KLRG1,ATXN1,IL1RL1,CRLF2,GATA3 |
| Immune | T cell | ILC | ILC2 | neg | CD4,FCER1A,IL12RB2,NCR2,NCR1 |
| Immune | T cell | ILC | ILC2 | sec | AREG,IL4,IL5,IL9,IL13 |
| Immune | T cell | ILC | ILC3 (NCR+) | pos | CCR6,IL2RA,PTPRC,THY1,IL7R,IL1R1,IL23R,NCR2,NCR1,ATXN1,RORC,AHR |
| Immune | T cell | ILC | ILC3 (NCR+) | neg | CD4,CD8A,CD8B |

| | | | | | |
|---|---|---|---|---|---|
| Immune | T cell | ILC | ILC3 (NCR+) | sec | CSF2,IL22,LTA,LTB,TNF |
| Immune | T cell | ILC | ILC3 (NCR-) | pos | CCR6,IL2RA,PTPRC,THY1,IL7R,IL1R1,IL23R,RORC,AHR,TBX21 |
| Immune | T cell | ILC | ILC3 (NCR-) | neg | NCAM1,NCR2,NCR1,ATXN1 |
| Immune | T cell | ILC | ILC3 (NCR-) | sec | CSF2,IL17A,LTA,LTB |
| Immune | T cell | ILC | LTI | pos | CCR6,CCR7,CD7,IL2RA,CD44,PTPRC,THY1,IL7R,KLRB1,CXCR5,CXCR6,IL1R1,IL2RG,IL23A,ITGA4,ITGB7,RORC |
| Immune | T cell | ILC | LTI | neg | CD4,IL12RB2,NCR1,NCR2 |
| Immune | T cell | ILC | LTI | sec | IL17A,IL22,LTA,LTB,TNF |
| Immune | T cell | NK cell | NK cell | pos | NCAM1,KLRD1,IL2RB,FCGR3A,KIR3DX1,KIR3DL1,KIR3DL2,KIR3DL3,KLRC1,KLRK1,NCR3,NCR2,NCR1,KLRF1,TBX21,EOMES |
| Immune | T cell | NK cell | NK cell | neg | CD3D,CD3E,CD3G,IL7R |
| Immune | T cell | NK cell | NK cell | sec | GZMB,IFNG,PRF1 |
| Immune | T cell | ILC | ILCreg | pos | IL2RA,PTPRC,THY1,IL2RB,IL7R,ATXN1,TGFBR1,TGFBR2,ID3,SOX4 |
| Immune | T cell | ILC | ILCreg | neg | CD4,KLRG1,NCR1,IL1RL1 |
| Immune | T cell | ILC | ILCreg | sec | IL10,TGFB1 |
| Immune | B cell | B cell | B cell (Follicular) | pos | CD19,MS4A1,CR2,CD22,FCER2,CD24,CD38,CXCR4,CXCR5,HLA-DRA,HLA-DRB1,HLA-DRB5,HLA-DRB3,HLA-DRB4,IGHD,IGHM,TNFRSF13B,PAX5 |
| Immune | B cell | B cell | B cell (Follicular) | neg | MME,CD27 |
| Immune | B cell | B cell | B cell (MZ) | pos | CD1C,CD19,MS4A1,CR2,FCER2,CD27,FCRL3,IGHD,IGHM,TNFRSF13B,EBF1,TCF3,POU2F2,PAX5 |
| Immune | B cell | B cell | B cell(Memory) | pos | CD80,CD86,CD19,MS4A1,CR2,FCER2,CD27,CD40,FAS,PTPRJ,TNFRSF13B,HLA-DRA,HLA-DRB1,HLA-DRB5,HLA-DRB3,HLA-DRB4,POU2AF1,PAX5,SPIB |
| Immune | B cell | B cell | B cell(Memory) | neg | CD38,CD93 |
| Immune | B cell | Plasma cell | Plasma cell | pos | TNFRSF17,CD27,CD38,CXCR4,HLA-DRA,HLA-DRB1,HLA-DRB5,HLA-DRB3,HLA-DRB4,SDC1,PRDM1,IRF4,XBP1,MZB1,JCHAIN |
| Immune | B cell | Plasma cell | Plasma cell | neg | MME,CD19,MS4A1,IGHD |
| Immune | B cell | B cell | B cell (Breg) | pos | CD1D,CD5,CD19,CR2,CD24,CD38,CD40,IGHM,EBF1,TCF3,POU2F2,PAX5,POU2AF1 |
| Immune | B cell | B cell | B cell (Breg) | sec | IL10,TGFB1,TGFB2,TGFB3,EBI3 |
| Immune | Myeloid cell | Macrophage | common | pos | CD80,CD86,CCR5,ITGAM,ITGAX,CD14,FUT4,CD68,CD163,ADGRE1,FCGR1A,FCGR1B,FCGR2A,FCGR2B,FCGR3A,FCGR3B,LGALS3,TNFRSF18,ITGAL,LAMP2,LILRB4,CSF1R,CD33,TLR2,TLR4,HLA-DMA,HLA-DMB,HLA-DOA,HLA-DOB,HLA-DPA1,HLA-DPB1,HLA-DQA1,HLA-DQA2,HLA-DQB1,HLA-DQB2,HLA-DRA,HLA-DRB1,HLA-DRB5,HLA-DRB3,HLA-DRB4 |
| Immune | Myeloid cell | Macrophage | Macrophage (M1) | pos | PTGS2,NOS2,IRF5,STAT1,CD80,CD86,CD36,CD68,FCGR2A,FCGR3A,IFNGR1,IFNGR2,HLA-DMA,HLA-DMB,HLA-DOA,HLA-DOB,HLA-DPA1,HLA-DPB1,HLA-DQA1,HLA-DQA2,HLA-DQB1,HLA-DQB2,HLA-DRA,HLA-DRB1,HLA-DRB5,HLA-DRB3,HLA-DRB4 |
| Immune | Myeloid cell | Macrophage | Macrophage (M1) | sec | CCL2,CCL3,CCL4,CCL5,CCL8,CCL11,CCL15,CCL19,CCL20,CXCL1,CXCL2,CXCL3,CXCL5,CXCL8,CXCL9,CXCL10,CXCL11,CXCL13,CXCL16,CX3CL1,IL1B,IL6,IL12A,IL12B,IL15,IL17A,IL18,IL23A,IFNG,TNF |

| | | | | | |
|---|---|---|---|---|---|
| Immune | Myeloid cell | Macrophage | Macrophage (M2A) | pos | IRF4,STAT6,PPARG,CD163,CD200,CLEC10A,CXCR1,CXCR2,CD209,CLEC7A,FCER1A,IL1R2,IL4R,MRC1,HLA-DMA,HLA-DMB,HLA-DOA,HLA-DOB,HLA-DPA1,HLA-DPB1,HLA-DQA1,HLA-DQA2,HLA-DQB1,HLA-DQB2,HLA-DRA,HLA-DRB1,HLA-DRB5,HLA-DRB3,HLA-DRB4 |
| Immune | Myeloid cell | Macrophage | Macrophage (M2A) | sec | CCL1,CCL2,CCL14,CCL17,CCL18,CCL22,CCL23,CCL24,CCL26,FIZ1,IL1RN,IL10,IL12A,IL12B,TGFB1,TGFB2,TGFB3 |
| Immune | Myeloid cell | Macrophage | Macrophage (M2B) | pos | PTGS2,IRF4,SOCS3,SPHK1,SPHK2,CD86,IL4R,HLA-DMA,HLA-DMB,HLA-DOA,HLA-DOB,HLA-DPA1,HLA-DPB1,HLA-DQA1,HLA-DQA2,HLA-DQB1,HLA-DQB2,HLA-DRA,HLA-DRB1,HLA-DRB5,HLA-DRB3,HLA-DRB4 |
| Immune | Myeloid cell | Macrophage | Macrophage (M2B) | sec | CCL1,CCL20,CXCL1,CXCL2,CXCL3,CSF3,CSF2,IL1B,IL6,IL10,TNF |
| Immune | Myeloid cell | Macrophage | Macrophage (M2C) | pos | IRF4,SOCS3,TLR8,CCR2,SLAMF1,CD163,IL4R,MRC1,MSR1,SCARB1,TLR1 |
| Immune | Myeloid cell | Macrophage | Macrophage (M2C) | sec | CCL16,CCL18,CXCL13,IL10,TGFB1,TGFB2,TGFB3 |
| Immune | Myeloid cell | Macrophage | Macrophage (M2D) | pos | NOS2 |
| Immune | Myeloid cell | Macrophage | Macrophage (M2D) | sec | CCL5,CXCL10,CXCL16,IL10,IL12A,IL12B,TNF,VEGFA,VEGFB,VEGFC,VEGFD |
| Immune | Myeloid cell | Monocyte | Monocyte | pos | ITGAM,CD14,CD163,CCR2,CCR5,CX3CR1,CSF1R,SELL,HLA-DRA,HLA-DRB1,HLA-DRB5,HLA-DRB3,HLA-DRB4,FCN1 |
| Immune | Myeloid cell | Dendritic cell | DC (Classical) | pos | CD1A,CD14,CD80,CD86,CCR7,CD1A,CD1C,ITGAM,ITGAX,CD40,CD83,IL3RA,THBD,CX3CR1,CLEC9A,CD209,EPCAM,CADM1,CD207,XCR1,HLA-DRA,HLA-DRB1,HLA-DRB5,HLA-DRB3,HLA-DRB4 |
| Immune | Myeloid cell | Dendritic cell | DC (Plasmacytoid) | pos | ITGAX,CLEC4C,IL3RA,NRP1,TCF4,IRF7,IRF8,SPIB,TLR7,TLR9,LILRA4,HLA-DRA,HLA-DRB1,HLA-DRB5,HLA-DRB3,HLA-DRB4 |
| Immune | Myeloid cell | Dendritic cell | DC (Plasmacytoid) | neg | CD1A |
| Immune | Myeloid cell | Dendritic cell | DC (Plasmacytoid) | sec | IFNA1,IFNB1,IL6,TNF |
| Immune | Myeloid cell | Dendritic cell | DC (Inflammatory) | pos | CD1A,CD1C,ITGAX,CD14,FCGR1A,FCER1A,MRC1,SIRPA,HLA-DRA,HLA-DRB1,HLA-DRB5,HLA-DRB3,HLA-DRB4 |
| Immune | Myeloid cell | Dendritic cell | DC (Inflammatory) | sec | IL23A,NOS1,TNF |
| Immune | Mast cell | Mast cell | Mast cell | pos | MITF,CD33,PTPRC,KIT,ENPP3,FCGR2A,FCGR2B,FCER1A,IL3RA,ITGAX,GATA2,TPSB2,TPSAB1 |
| Immune | Mast cell | Mast cell | Mast cell | neg | ITGAM,ITGA2 |
| Non-immune | Epithelial cell | Epithelial cell | Epithelial cell | pos | MUC1,EPCAM,DDR1,CADM4,MST1R,KLF5,KRT18,KRT19,CLDN1,CLDN3,CLDN4,CLDN12,CAPS,SNTN,CAPS,PIFO,TMEM190 |
| Non-immune | Endothelial cell | Endothelial cell | Endothelial cell | pos | CD300LG,CD34,CDH5,EGFL7,EMCN,ESAM,FLT1,FLT4,KDR,PODXL,S1PR1,SELE,SELP,TEK,THSD1,THSD7A,VCAM1,VEGFC,VWF |
| Non-immune | Fibroblast | Fibroblast | Fibroblast | pos | A2M,ACTA2,C1R,C1S,CADM3,CCDC80,COL1A1,COL1A2,COL3A1,COL5A1,COL6A2,ELN,FAP,FBLN1,FBLN2,LOXL1,LUM,PDGFRA,THY1,DCN,PLA2G2A,RARRES1 |

| | | | | | |
|---|---|---|---|---|---|
| Non-immune | Fibroblast | Fibroblast | Fibroblast | neg | EPCAM |
| Non-immune | Smooth muscle cell | Smooth muscle cell | Smooth muscle cell | pos | ACTA2,ACTG2,CALD1,CNN1,DES,EMILIN2,HEXIM1,HRH2,LGR5,LGR6,MYH11,MYL9,NOTCH3,TAGLN,MCAM,TPM2 |
